# ERK in Learning and Memory: A Review of Recent Research

**DOI:** 10.3390/ijms11010222

**Published:** 2010-01-13

**Authors:** Sheng Peng, Yan Zhang, Jiannan Zhang, Hua Wang, Bingxu Ren

**Affiliations:** Department of Anesthesiology, Affiliated No.4 Hospital of Soochow University, Wuxi, 214062, China; E-Mails: shengpeng@hotmail.com (P.S.); Jiannan.Zhang@hotmail.com (J.Z.); huan-wang@live.cn (H.W.); bxren@live.com (B.R.)

**Keywords:** ERK, learning and memory, neurobiology

## Abstract

The extracellular signal-regulated kinase (ERK) pathway is a member of the mitogen-activated protein kinase (MAPK) superfamily, which is an important, highly conserved family of enzymes associated with cell membrane receptors and regulative targets. In the central nervous system, there is almost no mature neuronal proliferation and differentiation, but the regulation of MAPK and its upstream and downstream molecular pathways is still widespread, with the ERK signaling pathway being one of the most actively studied signal transduction pathways. It is activated by a variety of cell growth factors and substances which promote mitotic activity, and transmits extracellular signals from the cell surface to the nucleus, which transmission plays an important role in the process of cell proliferation and differentiation. In recent years, accumulating evidence has shown that the ERK signaling pathway has an important link with the higher functions of learning and memory.

## 1. Introduction

Learning and memory are high-level brain functions, and they constitute an area of rapid advances in the field of neurobiology. At present, the neurobiology of learning and memory is largely focused on the high degree of plasticity in the central nervous system, especially synaptic plasticity and its functions. Synapses are a critically important structure for the transmission of information between neurons. Under certain conditions, the effectiveness of synaptic transmission depends upon morphological change to modify biological function. The process of central excitatory synaptic transmission, including both long-term potentiation (LTP) and long-term depression (LTD), is a well-known model for neural synaptic plasticity research.

The extracellular signal-regulated kinase (ERK) pathway belongs to the mitogen-activated protein kinase (MAPK) superfamily, which is an important, highly conserved family of enzymes associated with cell membrane receptors and regulative targets. In the central nervous system, there is almost no mature neuronal proliferation and/or diferentiation, but the regulation of MAPK and its upstream and downstream molecular effectors is still widespread. The ERK signaling pathway is one of the most actively studied these signal transduction pathways. It is activated by a variety of cell growth factors and substances promoting mitotic activity, and transmits extracellular signals from the cell surface to the nucleus, which transmission plays an imortant role in the process of cell proliferation and differentiation. In recent years, accumulating evidence has shown that the ERK signaling pathway has an important link with the functions of learning and memory [1–4].

## 2. ERK in Learning and Memory

### 2.1. ERK

The MAPK cascade signaling pathway is a critically important cell-mediated response. The composition of the MAPK pathway and its upstream regulating kinase form a functional unit, serving as a bridge between the upstream input signal and a wide variety of output signals. The functional unit is composed of three kinds of kinases, MAPK kinase kinase (MKKK), MAPK kinase (MKK), MAPK. These allow a series of kinase cascades which constitute a module for the activation of the three components. The module from yeast to humans is very highly conserved. MKKK is a serine/threonine kinase, and it effects the phosphorylation of MKK when it is activated; MKK is a dual specificity kinase that recognizes and phosphorylates threonine-X-tyrosine (Thr-X-Tyr) MAPK activity. It activates the Thr-X-Tyr sequence, in which X can be any of the eukaryotic amino acids. MAPK is the last kinase of the activated three-component module, and the MAPK phosphorylation site is the substrate of the serine and threonine residues. The MAPK pathways in mammalian cells is divided, based on the different Thr-X-Tyr sequences, into the ERK, c-Jun N-terminal kinase (c-Jun *N-terminal* kinase, JNK)/stress-activated protein kinase (stress actived protein kinase, SAPK), p38MAPK and ERK5/BMK1 (big MAP kinase1) subfamily. The above MAPKs in the protein kinase VIII subfamily of the threonine and tyrosine sites activate different motifs. ERK is a threonine-glutamic acid-tyrosine (Thr-Glu-Tyr) motif [5,6]. ERK is divided into two subtypes: ERK1 and ERK2.

It is generally believed that the activation of ERK signaling takes place via the following four pathways [7–10]: (1) Receptor tyrosine kinase stimulation of Ras activation: growth factor → receptor tyrosine kinase → Ras → MAPKKK → MAPKK → ERK. (2) Ca^2+^ stimulated Ras activation: Ca^2+^ activates Ras by various mechanisms. Ca^2+^ flows into the cells through the L-type voltage-dependent calcium channel, and through the Src-mediated protein kinase family, leading to the phosphorylation of the epidermal growth factor (EGF) receptor tyrosine kinase, and further activation of Ras through the Shc-Grb-Sos complex. (3) Protein Kinase C (PKC) activation of the ERK pathway: PKC regulates the activity of ERK isozymes by different mechanisms, and this regulation is specific to cell type. The PKC agonist phorbol esters stimulate T cells and B cells, which can activate ERK. This is Ras-dependent, but activation of ERK in fibroblasts with phorbol esters is Ras-independent. (4) G protein-coupled receptor activation of the ERK pathway: guanine nucleotide binding protein (G protein)-coupled receptors activation of ERK through both Ras-dependent and Ras-independent pathways.

### 2.2. ERK with LTP and Synaptic Plasticity

It is generally accepted that long-term potentiation (LTP) is one of the cellular mechanisms involved in learning and memory. In 1997, England found that the application of the MEK inhibitor PD98059 inhibited hippocampal LTP, the first evidence of a role of ERK signaling in synaptic plasticity. In 2001, Dicristo and his collegues [11] reported that the participation of cortical neurons in the LTP pathway also requires the activation of ERK. Subsequenty Gooney *et al*. [12] observed that brain-derived neurotrophic factor (BDNF) = induced LTP also requires the activation of ERK. A number of behavioral experiments further confirmed that ERK is involved in the process of learning and memory. Blum *et al*. [13] reported that water maze training in mice can lead to the activation of ERK in the hippocampus. Selcher *et al*. [14] reported that PD98095 administered by injection into the mouse hippocampus or systemic drug delivery, led to the weakening of memory in animals by the activation of the inhibitor SL327 and of the blood-brain barrier potent ERK. In an offensive odor avoidance experiment, Bearman *et al*. [15] observed that the ability of rats to learn a new odor associated with the avoidance response was weakened by MEK blockers. Selcher *et al*. [16] found that starting from 1 on the frequency of 100 Hz induced by tetanic stimulation induced LTP, which required the activation of ERK in rats, but not mice. However, an ERK inhibitor blocked endogenous θ wave generation of LTP in these animals. The above experimental results show that ERK activation, LTP and learning and memory have close links with each other.

Recent investigation has demonstrated that the Ras/ERK pathways are involved in the processes of human cognition. Silva *et al*. [17] found learning decrements to be closely related to deficits in the Ras/ERK signaling pathway in an investigation of the relationship between neurofibromatosis type I blastoma (NF1) and mental retardation that was conducted utilizing genetic engineering technology. The NF1 gene product is the neurofibromatosis protein (neurofibromin), a multi-domain molecule regulating a variety of intracellular pathways, which can activate the ERK pathway by means of Ras GTP synthase. Following the genetic NF1 mutation in certain types of nerve fiber blastoma, GAP is compromised, resulting in excessive Ras activity in the hippocampus, causing the Ras/ERK pathway to become further over-excited, resulting in a decrease of learning and memory [18,19]. Costa *et al*. [20] also reported that precise neurofibroma protein regulation and control of Ras is necessary for cognition, and that NF1 gene mutation in mice caused a partial deficiency of the neurofibromatosis protein such that the activity of Ras was significantly increased, with the result that the inhibitory function of the GABA-mediated signal was abnormally enhanced, which can directly damage synaptic plasticity, thus giving rise to learning deficits.

### 2.3. The Impact of ERK on the Nuclear Transcription Factors Involved in LTP and Synaptic Plasticity

In a similar manner as observed in non-nerve cells, the activation of ERK in mature neurons results in a translocation from the cytoplasm into the nucleus [21,22], suggesting that the regulation of transcription factors may be one of the roles of the ERK signaling system. Davis, *et al*. [23] found that LTP in the process of the dentate gyrus of the hippocampus was induced along with the phosphorylation of the transcription and activation of the transcription factor Elk1 was sensitive to a MAPK/ERK kinase inhibitor. In a study of invertebrates and rodents, Lonze *et al*. [24] found that the transcription factor CREB has an important impact on various forms of learning and memory. Shaywitz, *et al*. [25] reported that the phosphorylation of the Ser133 residue of cAMP response element binding (CREB) had an impact on the regulation of gene transcription.

Hardingham *et al*. [26] found that MAPK/ERK kinase inhibitors block the sustained phosphorylation of CREB residue Ser133 and exert an effect the process of the CREB-dependent transcription, suggesting that ERK has a role in the regulation of CREB activity. Xing *et al*. [27] have shown that RSKs (ribosomal protein S6 kinases) and MSKs (mitogen and stress-activated protein kinases) downstream of ERK induced CREB-Ser133 as part of its substrate *in vitro*. Trivier *et al*. [28] found that RSK2 gene mutations induce learning deficits in mice and humans. The above results suggest that ERK plays a regulatory role in the process of LTP, as well as learning and memory, through the activity of certain nuclear transcription factors.

### 2.4. ERK along with LTP and Synaptic Plasticity Are Related to the Protein Translation

From the time course data, LTP can be divided into different phases utilizing different molecular mechanisms, respectively. The first approximate 30 min is termed short-term potentiation (STP). This stage is suggested to be related to the processes of resting receptors on the postsynaptic membrane, and to have nothing to do with the role of protein kinases. The next 1–2 h is known as E-LTP (early-LTP), and is closely related to the role of protein kinase phosphorylation of the receptor, such as the CaMK II effect on the α-amino-3-hydroxy-5-methyl-4-isoxazolepropionic acid (AMPA) receptor. The L-LTP (late-LTP) phase requires new protein synthesis, as well as the involvement of PKA. Studies have found that the ERK signaling system has an effect on not only postsynaptic receptors and nucleoprotein-related transcription factors, but also a regulatory effect on LTP through translation of the protein [29–31].

Kelleher *et al*. [32] observed a learning and memory association of MAPK and synaptic plasticity with the effects of protein synthesis in dnMEK1 mice (dominant-negative MEK1 mice). This dnMEK1 mouse lacks the activity of mitogen-activated protein kinase 1 (MEK1) kinase, but retains the capacity for the combiNation with ERK1/2. In a study of LTP in hippocampal slices, it was found that synaptic transmission and the function of E-LTP were normal, but L-LTP was selectively weakened. This may be because the phosphorylation levels of the protein translation of the initiation factor in the hippocampal neurons had been reduced, thus weakening the selective formation of LTP involved in protein synthesis.

In the initiation of protein translation, the initiation factor elF4E recognizes the mRNA 5′ terminal cap structure, followed by the combiNation of the elF4G subunit with ribosomal 40S. This process is considered to be a key initial step. eIF4E phosphorylation and the elF4E inhibitor 4E-BP1 modify the ability of elF4E to recognize the mRNA 5′ terminal cap structure and also the binding with elF4G. Kelleher *et al*. [32] found the elF4E and S6 ribosomal proteins to be highly dependent on the form of ERK phosphorylation, and similar structures involved in the process of the elF4E and S6 ribosomal protein phosphorylation in the case of hippocampal LTP also require the activity of ERK. This suggests that ERK plays an important role in the formation and maintenance of LTP at the protein translation level.

The θ frequency stimulation can mimic rodent hippocampal CA1 neuronal discharge patterns in the learning environment. It reportedly induces the action potential in the hippocampal CA1 region, and then backpropagates to dendritic synapses, resulting in the depolarization. Winder *et al*. [22] found that a block of ERK activation in rat hippocampal CA1 inhibited θ frequency stimulation-induced LTP. Winder *et al*. [33] found that the θ frequency stimulation which induced LTP by the β-adrenergic receptor was also inhibited by ERK activation blockers. Gelinas *et al*. [34] observed hippocampal CA1 area-induced L-LTP when β-adrenergic receptor activation and subthreshold electrical stimulation was applied. In this case, only the translation and synthesis of the new protein was observed at the dendritic sites, not transcription in the cell body, and this form of LTP also requires the activation of ERK. The above study also suggested a key role LTP in association with protein synthesis.

### 2.5. ERK Affect on NMDA and AMPA Receptors

In the course of LTP induction, the (*N*-methyl-d-aspartate) NMDA receptor and Ca^2+^ play a critical role. After the Voltage-ligand-gated NMDA receptor is activated, it mediates Ca^2+^ entry into the cell and activates a number of biochemical pathways. It also triggers a series of reactions which result in the increase of synaptic responses and the formation of LTP. The activation of the NMDA receptor is a key step in NMDA receptor-dependent hippocampal LTP induction, and the depolarization of postsynaptic membrane is one of the necessary conditions for the activation of the NMDA receptor.

PKA, PKC, and β-adrenergic receptor activators regulate the neuronal dendritic K^+^ channel through the activation of ERK, and the Kv4 family of dendritic voltage-dependent K^+^ channels controls the degree of the synaptic depolarization by regulating the depolarization levels of their cell membranes, which further affects the induced NMDA receptor-dependent LTP [35,36]. The activation of ERK leads to the phosphorylation of the K^+^ channel α-subunit of the Kv4 family, which apparently changes the K^+^ channels from being voltage-dependent to depolarization-dependent. This increases the excitatory state of the synaptic membrane and opens the K^+^ channels, leading to an even stronger depolarization. Increase in the excitability of the membrane results in a stronger postsynaptic membrane depolarization mediated by the AMPA receptor-inward current, and ultimately leads to the activation of NMDA receptors [37,38]. This depolarization affects other synaptic neurons, such that LTP is facilitated. An increase in the number of AMPA-type glutamate receptors in the postsynaptic membrane and enhancement of receptor activity play an important role in the process of LTP generation. Some synapses in the resting state exhibit only an NMDA receptor reaction, not a reaction of the AMPA receptor. When LTP is induced or CaMK II activated, the AMPA receptor appears in the postsynaptic membrane, so that the effectiveness of synaptic transmission is evidently increased. In a study of LTP induction and maintenance in hippocampal slices, Zhu *et al*. [39] observed a relationship between the Ras-ERK cascade signaling pathway and the AMPA receptor, and also with synaptic plasticity, through the activation of recombinant AMPA receptors. Their results showed that the activity of Ras increased the response of synaptic AMPA receptors, and this effect was blocked by the MAPK/ERK kinase inhibitor PD98095, suggesting that the ERK activation in this process is associated with the activity of the AMPA receptor. In addition, the activation of CaMK II mediation of the enhanced AMPA receptor was equally sensitive to PD98095, suggesting that ERK plays a regulatory role in the process of the CaMK II-mediated AMPA receptor insertion into the postsynaptic membrane. Hence, it is speculated that the role of CaMK II in hippocampal LTP may be effected through Ras proteins, and thus lead to ERK activation. The AMPA receptor insertion into the postsynaptic membrane would be likely to increase the size of the dendritic spines or to induce the development of new pseudopods (filopodia) and dendritic spines, thereby enhancing the effectiveness of synaptic transmission. As the neuronal dendrites comprise 90% of the total surface area, changes in the dendrite number, length and cross-sectional area are closely related with changes in brain function. It is reported that the MEK inhibitor U0126 not only blocks the LTP of hippocampal neurons *in vitro*, it also blocks the formation of new dendritic spines and pseudopods [40,41]. These results suggest that ERK affects the morphology of synaptic plasticity in the regulation of the insertion of the AMPA receptor into the postsynaptic membrane, and furthermore, plays a regulatory role in neural synaptic transmission [42].

### 2.6. The ERK Pathway and Long-Term Memory

In a study by Kelly *et al*. [43], DMSO (control group) and the MAPK/ERK inhibitor UO126 (experimental group) were injected into the ventricle of SD rats. The use of behavior monitoring methods revealed that the time spent searching for new and old targets after 40 minutes of training was not significantly different between the groups *(P >* 0.05). However, on the second day, the time required by the experimental group to find both new and old targets was significantly longer than the control group *(P* < 0.01). The results showed that there was an effect on the maintenance of long-term memory and that the learning function was blocked, but there was no significant effect on short-term memory by the administration of UO126 in rats. Mazzucchelli *et al*. [44] observed that ERK1 knockout in the striatum affected both learning and memory in a study of one-trial passive avoidance behavior in mice. The results showed that ERK1 knockout mice did not exhibit an affect after the first 30 minutes of training, but after the first 24 hours of training, in the knockout mice the memory was enhanced, and the avoidance before entry into the dark was significantly prolonged *(P* < 0.01). These results suggest that long-term memory function was significantly improved and short-term memory retained in the ERK1 knockout mice. Zhang *et al*. [45] found that PD98059 damaged olfactory function as well as learning and memory in young rats after injection into the olfactory bulb in rats, but this did not affect the memory displayed after 1 h of training. Western blot analysis confirmed that the phosphorylation of ERK1 and ERK2 was significantly increased after odor shock training for 1h in normal rats, and thus activated the ERK pathway, and PD98059 significantly decreased the levels of ERK phosphorylation, which affected the function of learning and memory in rats.

### 2.7. ERK Signaling Pathway with Other Pathways of Interation

The crucial functions of learning and memory are associated with complex physiological and biochemical mechanisms, including the ERK pathway, in association with a number of other signal transduction pathways. Studies have confirmed that certain learning and memory functions are associated with interactions among the GABA pathway, ERK, the cholinergic signaling pathway and their reciprocal interactions. The generation of LTP usually requires the activation of the NMDA receptor, with this process mainly regulated by the Kv4-encoded A-type potassium ion channel family members [46]. Yuan and Morozov *et al*. [47,48] found that the activation of the hippocampal CA1 β-adrenergic receptor, as well as the PKA and PKC activation cascades, induced the phosphorylation of Kv4.2α subunit by the activation of ERK, a process which was blocked by the ERK inhibitor UO126 or PD98059. The phosphorylation of Kv4.2 α caused a reduction of the A-type K^+^ current, and induced amplification of the action potential (AP), and thereby enhanced the excitability of the dendritic membrane. Studies have also reported that after blocking the MAPK/ERK pathway with ERK inhibitors, the activation of the β-adrenergic and cholinergic receptors, which induce the formation of LTP, was inhibited, and the generation of LTP with 5 Hz of electrical stimulation was also inhibited [49]. In the latest report by Watanabe *et al*. [50], dendritic membrane potassium channels were shown to be regulated by the PKA and PKC signaling cascades as well as β-adrenergic receptor activation, and this regulation depended on the activation of ERK. In addition, PKA-induced Rap-1 binding with B-Raf, which led to the phosphorylation of MAPK/ERK and the activation of cAMP response element binding protein (CREB), which participated in the formation of PKA-dependent LTP and the changes of synaptic plasticity [51]. In a study of the relationship between histone acetyltransferase (HAT) and learning and memory [52], Swank and his colleagues found that the ERK cascade pathway regulated the acetylation of a 42KD substrate of lysine acetyltransferase, indicating that there is a causal relationship between the activation of ERK and HAT activity in the cerebral cortex.

## 3. Conclusions

The MAPK cascade signaling pathway is of critical importance in a broad variety of cells. It responds to a wide range of extracellular stimuli, and is a major mechanism by which extracellular signals are transduced in the cell. The activation of ERK1/ERK2 regulates synaptic proteins and promotes the formation of new dendritic spines. It also has an impact on the phosphorylation status of nuclear transcription and translation factors related to LTP and synaptic plasticity, and plays a positive regulatory role in the induction and maintenance of LTP. Various studies have demonstrated a number of pathways which participate in the induction and maintenance of LTP, but a clear and detailed description of the interaction between the various signaling pathways and the regulation of LTP awaits further investigation.
